# Development and Characterization of Gelatin-Based Hydrogels Containing Triblock Copolymer and Phytic Acid

**DOI:** 10.3390/gels10050294

**Published:** 2024-04-25

**Authors:** Njomza Ajvazi, Ingrid Milošev, Romana Cerc Korošec, Peter Rodič, Bojan Božić

**Affiliations:** 1 Jožef Stefan Institute, Jamova 39, 1000 Ljubljana, Slovenia; njomza.ajvazi@ijs.si (N.A.); peter.rodic@ijs.si (P.R.); bbozic@bio.bg.ac.rs (B.B.); 2 Valdoltra Orthopaedic Hospital, Jadranska c. 31, 6280 Ankaran, Slovenia; 3 Faculty of Chemistry and Chemical Technology, University of Ljubljana, Večna Pot 113, 1000 Ljubljana, Slovenia; romana.cerc-korosec@fkkt.uni-lj.si; 4 Faculty of Biology, Institute of Physiology and Biochemistry “Ivan Djaja”, University of Belgrade, Studenski Trg 3, 11000 Beograd, Serbia

**Keywords:** hydrogels, gelatin, Pluronic F-127, phytic acid, biomolecules

## Abstract

In recent research, significant interest has been directed towards gelatin-based hydrogels due to their affordable price, extensive availability, and biocompatibility, making them promising candidates for various biomedical applications. The development and characterization of novel hydrogels formed from varying ratios of gelatin, triblock copolymer Pluronic F-127, and phytic acid have been presented. Swelling properties were examined at different pH levels. The morphology of hydrogels and their thermal properties were analyzed using scanning electron microscopy (SEM), thermogravimetric analysis (TG), and differential scanning calorimetry (DSC). Fourier-transform infrared (FTIR) analysis of the hydrogels was also performed. The introduction of phytic acid in the hydrogel plays a crucial role in enhancing the intermolecular interactions within gelatin-based hydrogels, contributing to a more stable, elastic, and robust network structure.

## 1. Introduction

Hydrogels, often introduced as polymeric mixtures, possess hydrophilic characteristics [1] that enable the retention of water and biofluids [2,3]. These three-dimensional polymeric structures have significant roles in diverse applications, including wound healing, contact lenses, tissue engineering, implant coating, agriculture, bio-sensors, drug delivery, and various hygiene products [4]. Hydrogels can swell in water without dissolving, a phenomenon facilitated by cross-linking within their network chains, allowing them to retain water in their structure without undergoing dissolution [4,5,6,7].

Gelatin is a commonly studied and prominent example of hydrogel preparation. Derived from collagen degradation, gelatin is a widely utilized ingredient for both food and non-food applications. Renowned for its non-toxic and biodegradable attributes, gelatin serves diverse purposes, including promoting gelation, stabilizing, thickening, emulsifying, and forming films [8]. Gelatin, as a natural biopolymer, possesses a significant ability to form hydrogels with various compositions, facilitating effective cross-linking. Gelatin-based hydrogels are a specific type of hydrogel that utilize gelatin as the cross-linked polymer, which confer specific structural and textural characteristics to the gel [9]. Following its applications, gelatin-based hydrogels can readily biodegrade, making them suitable for sustainable use in the environment. Moreover, the biodegradability of these hydrogels highlights their eco-friendly nature, contributing to the reduction of environmental waste [10,11]. However, it is essential to combine gelatin with other eco-friendly materials to improve the hydrogel’s structural characteristics. To achieve novel polymers through gelatin-based hydrogels, it is crucial to consider the ratio between the components, reaction time, reaction temperature, and the drying of the material. These factors play a key role in preventing denaturation of gelatin macrostructure and ensuring the hydrogel’s stability and gradual degradation [9].

Gelatin has drawbacks in terms of mechanical characteristics, displaying fragility and susceptibility to fractures. Moreover, these mechanical limitations of gelatin may hinder its suitability for applications that demand durability and resistance to breaking. The chemical modification of gelatin through cross-linking involves the reaction of chemical reagents with active groups on the gelatin chain to create new chemical bonds, providing a network structure that modifies its properties for various applications [12]. Physically cross-linked gelatin-based hydrogels are typically formed through reversible intermolecular interactions, which are characterized by non-covalent bonding. The primary advantage of physical cross-linking is the absence of chemical cross-linking agents, thereby reducing the risk of cytotoxicity from unreacted chemical cross-linkers [13,14].

Pluronic F-127 is a surface-active tri-block copolymer featuring a central hydrophobic (polypropylene oxide) chain and two outer hydrophilic poly (ethylene oxide) chains. Pluronic F-127 is FDA approved and listed in the U.S. and European Pharmacopoeia [15,16]. This polymer shows amphiphilic properties [17], and, owing to its non-toxicity, water-solubility, biocompatibility, and sol–gel properties sensitive to heat, is desirable for various biomedical applications [18]. The use of nanocarriers enhances the effective transportation of drugs and allows for continuous and prolonged drug release. These hydrogels, incorporating drug-loaded nanocarriers, protect the drug from degradation and prolong its residence time at the administration site [16]. Several methodologies have been developed involving the cross-linking of Pluronic F-127 with different origine polymers, such as alginate [19], chitosan [20], gellan gum [21], types of gelatin (type A, GA, and type B, GB) [22], gelatin–lecithin [23], etc.

Phytic acid (PA), as the predominant inositol phosphate in nature, is the major component of plant seeds [24] and plays an essential role in various biological functions. It exhibits distinctive properties, with a high negative charge density conferring significant chelating ability and valuable antioxidant properties [25]. Beyond its antioxidant and antibacterial effects, PA can potentially enhance the biological properties and stability of hydrogels without introducing toxicity [24]. PA stands out as a potential candidate for naturally cross-linking scaffolds due to its abundant hydroxyl-bearing phosphoric groups. In the cross-linking process, PA anions bond with cations of natural polymers, and hydrogen bonds form through the hydroxyl groups in the compound’s structure [26]. The findings substantiate that PA resulted in a hydrogel scaffold exhibiting enhanced mechanical properties and antimicrobial capability, making it a promising candidate for various biomedical applications [27].

Numerous methodologies have been developed involving the cross-linking of PA with various polymers, including polyaniline [28], chitosan [29], poly((trimethylamino)ethyl methacrylate chloride) [27], poly(vinyl alcohol) [30], alginate [31], gelatin/polycaprolactone [26], polyacrylamide/chitosan [32], and carboxymethyl cellulose [24], aimed at using hydrogels for diverse biomedical applications. Choosing naturally derived cross-linking agents not only ensures safety for biomedical applications but contributes to both scientific and environmental goals in medical advancements. This emphasis on natural sources underscores a broader commitment to eco-friendly practices in the pursuit of innovative biomedical solutions.

Despite the use of toxic crosslinkers and catalysts as essential components typically used in hydrogel preparation, this study presents a novel protocol that combines gelatin, Pluronic F-127, and phytic acid to deliver a unique hydrogel network. The goal is to provide sustainable and safe solutions for various biomedical applications. Notably, this network is non-toxic, environmentally friendly, and biodegradable.

## 2. Results and Discussion

**Figure 1:**
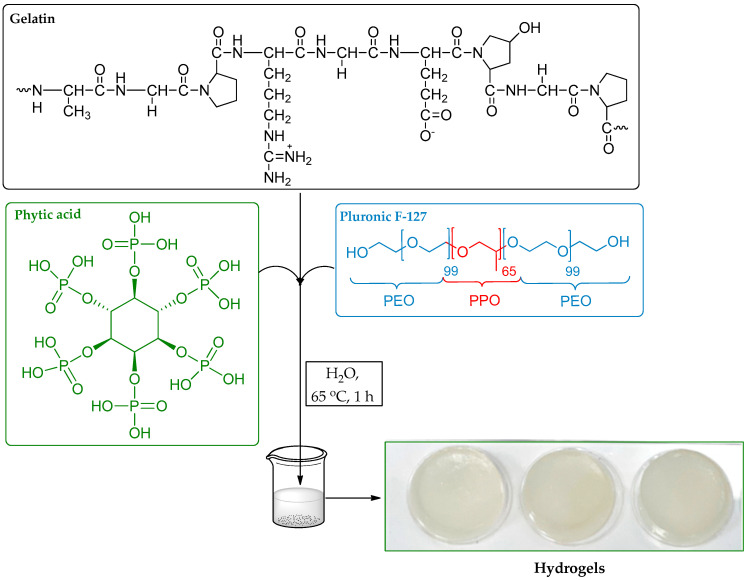
Flowchart of hydrogel preparation.

Various hydrogel samples were prepared by adjusting the proportions of gelatin (G), Pluronic F-127 (F-127), and phytic acid (PA) in the mixture, as outlined in Table 1 and Figure 1. The mixture was also prepared without PA (entry 4, Table 1).

**Figure 2:**
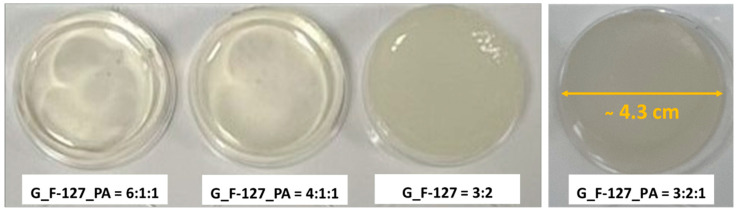
Hydrogels were obtained after cross-linking for 24 h at room temperature.

Various formulations of freshly prepared hydrogels are illustrated in Figure 2 after cross-linking for 24 h at room temperature. The absence of PA (G_F-127 = 3:2) results in a hydrogel that is not clear, lacks smoothness, and has a low degree of flexibility. Adding PA (G_F-127_PA = 6:1:1 and G_F-127_PA = 4:1:1) makes the hydrogel clear and smooth. In particular, the higher amounts of F-127 and PA (G_F-127_PA = 3:2:1) have been shown to enhance the smoothness and flexibility of the hydrogel in comparison to other formulations.

### 2.1. FTIR Spectroscopy Analysis

**Figure 3:**
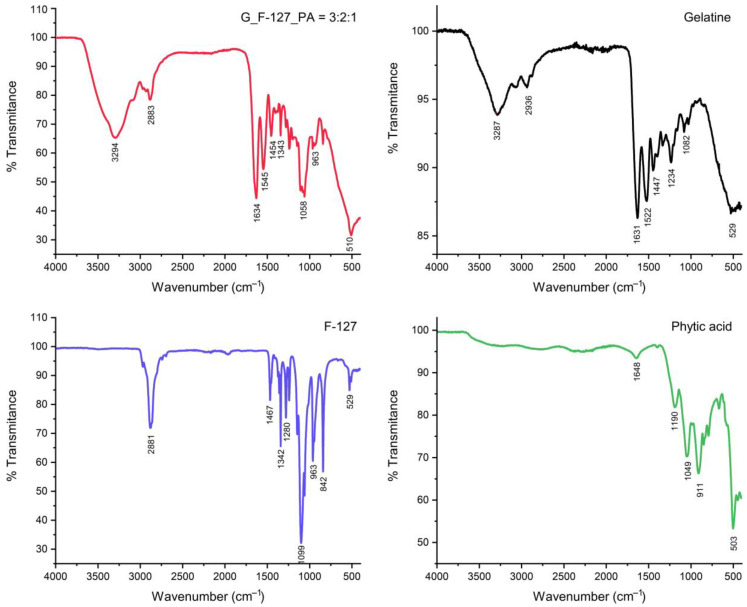
FTIR spectra of the hydrogel G_F-127_PA = 3:2:1 and initial reagents (G, F-127, and PA).

Figure 3 shows the FTIR spectra of the pure gelatin, F-127, PA, and the optimized hydrogel (G_F-127_PA = 3:2:1). The IR spectra of the G_F-127_PA = 3:2:1 structure reveal characteristic bands from its constituent components, all of which contribute to the formation of the hydrogel network. A notable observation is a shift to a higher wavelength of the broad band at ca. 3330 cm^−1^ regions, which is attributed to the OH and NH_2_ vibration. Furthermore, the intensity of the band corresponding to the C=O group at 1625 cm^−1^ originating from gelatine was observed in the hydrogel spectrum. Additionally, the band of P-O groups at 1188 cm^−1^ in PA shifts to a higher wavelength, 1239 cm^−1^, in the hydrogel, indicating the formation of intermolecular bonds between gelatin and/or F-127 with the PA.

### 2.2. Swelling Test

**Figure 4:**
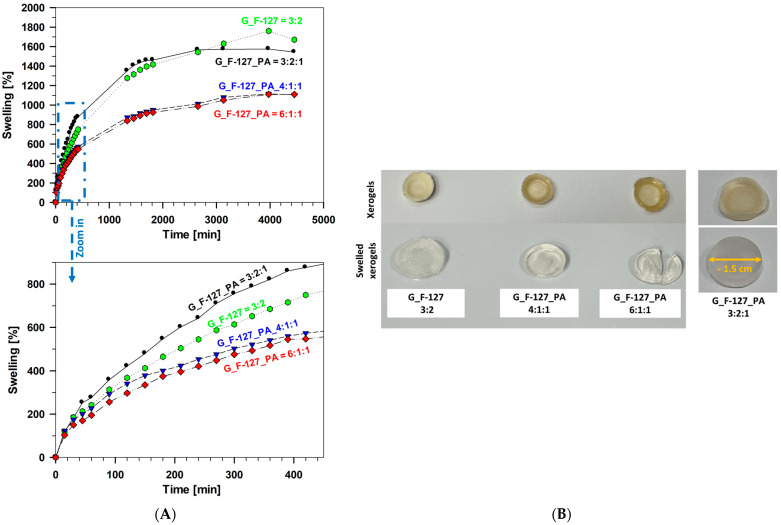
(**A**) The swelling percentages of the investigated hydrogel samples: G_F-127 = 3:2, G_F-127_PA = 3:2:1, G_F-127_PA = 4:1:1, and G_F-127_PA = 6:1:1 in phosphate buffer solution (PBS, 0.1 M) at a pH of 7.4. (**B**) Images of swollen xerogels in a 0.1 M PBS buffer at 30 °C after 74 h.

The swelling percentage of all investigated hydrogel samples over time in phosphate-buffered saline solution (PBS, 0.1 M) at a pH of 7.4 is presented in Figure 4A. The degree of swelling depends on the amount of cross-linkers, specifically on the ratio between the contents of G, PA, and F-127. Lower absorption is observed as the amount of cross-linkers decreased (G_F-127_PA = 6:1:1 and G_F-127_PA = 4:1:1). The highest degree of swelling was observed in sample G_F-127 = 3:2 in the terminal period of swelling (1671%) and exhibited a slightly greater degree of swelling than sample G_F-127_PA = 3:2:1 (1544%). More importantly, sample G_F-127_PA = 3:2:1 exhibited a faster rate of swelling (Figure 4A, zoom part). In all cases, signs of saturation were observed, reaching a plateau with a decrease in swelling rate after two days. The higher incorporation of F-127 and the PA afforded compact interaction of the polymer network, as confirmed by SEM (see Section 2.3.). These porous surfaces allow water to move easily through the polymeric structure, thereby increasing the swelling capacity of the hydrogels.

After drying to a constant weight at ambient temperature for seven days, hydrogel samples were transformed into xerogel (Figure 4B, upper row). After the swelling process was performed in PBS buffer for 74 h (ca. 3 days), the xerogels swelled (Figure 4B, lower row). It appears that an increased quantity of F-127 led to a lower degradation rate, as observed in sample G_F-127 = 3:2. Furthermore, the samples G_F-127_PA = 4:1:1 and G_F-127_PA = 6:1:1 exhibited weakened structures during the swelling process, making them highly susceptible to rupture upon handling. The hydrogels containing increased amounts of both F-127 and PA (G_F-127_PA = 3:2:1) were the most stable, fully retaining a cylindrical shape.

**Figure 5:**
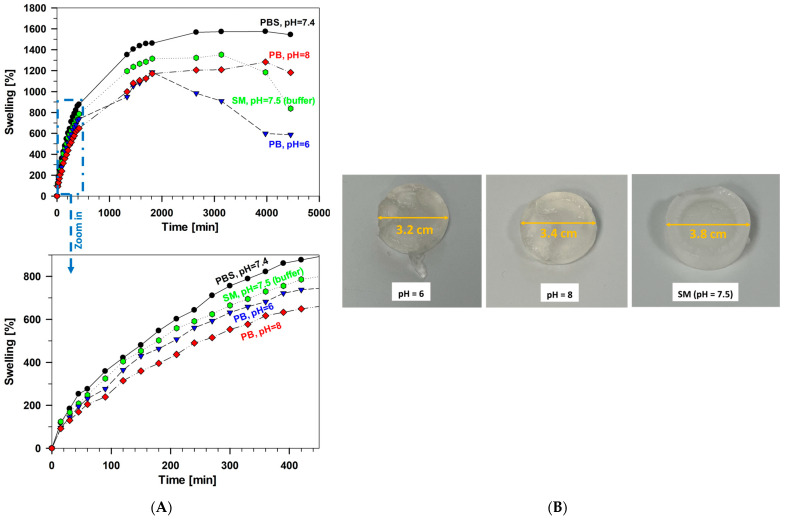
(**A**) The swelling percentages of the sample G_F-127_PA = 3:2:1 at PBS, different pHs of phosphate buffer (PB), and SM solutions. The lower graph is an enlargement of the initial 400 min in the upper graph. (**B**) Xerogel samples G_F-127_PA = 3:2:1 after 4 days of swelling in phosphate buffer solutions at pH 6 and pH 8 and SM at pH 7.5 at 30 °C.

Moreover, the investigated samples exhibit different absorption capacities depending on the pH changes of the environmental solution (Figure 5A). The hydrogel G_F-127_PA = 3:2:1 showed the highest swelling degree at pH 7.4 and contracted under more acidic conditions of phosphate buffer solution at pH 6 and in SM buffer (a mixture of NaCl, MgSO_4_, Tris-HCl, and gelatin, pH 7.5). These changes in swelling behavior were attributed to the pH sensitivity of the hydrogel’s main components: gelatin, F-127, and phytic acid. Consequently, distinct swelling patterns were observed based on the specific pH conditions (Figure 5B).

### 2.3. Scanning Electron Microscopy (SEM) Investigation

**Figure 6:**
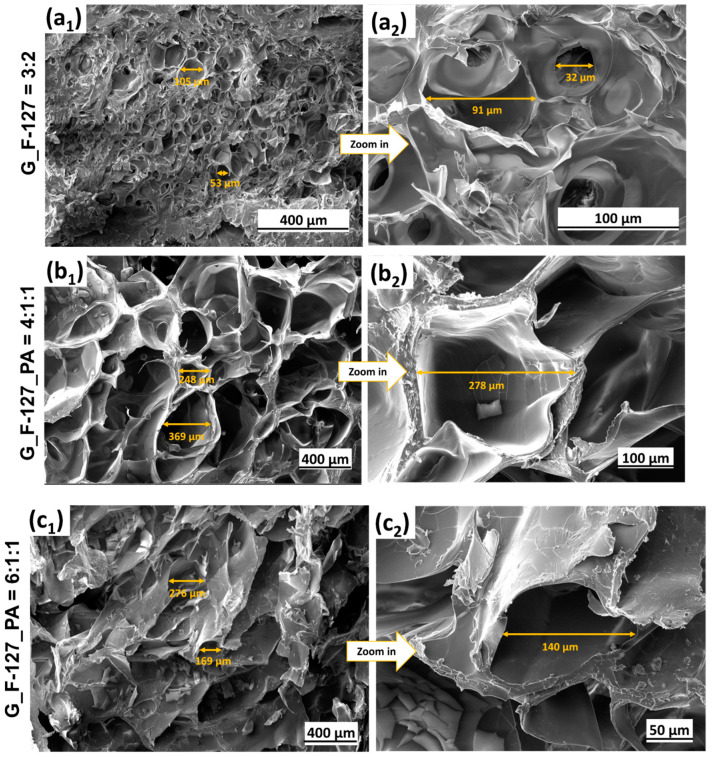

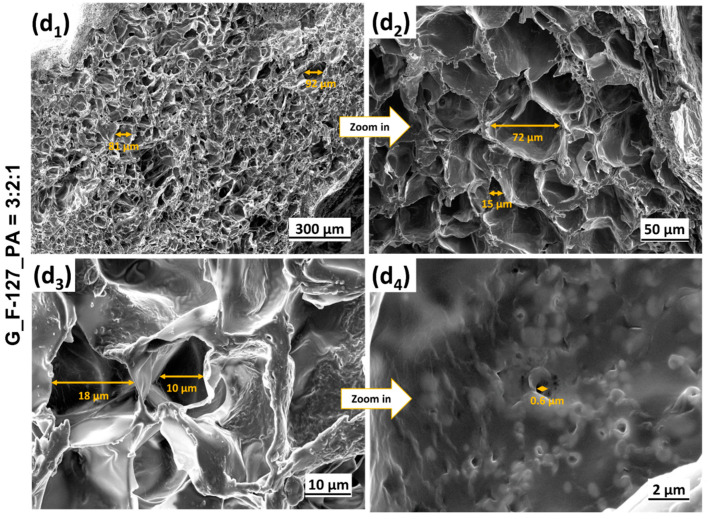
Secondary electron (SE) SEM images of lyophilized samples: (**a1**,**a2**) G_F-127 = 3:2; (**b1**,**b2**) G_F-127_PA = 4:1:1; (**c1**,**c2**) G_F-127_PA = 6:1:1; and (**d1**–**d4**) G_F-127_PA = 3:2:1.

Investigated samples made by different ratios of G_F-127_PA components have been lyophilized, and recorded and analyzed by SEM (Figure 6). The size and organization of pores differ between the samples. The measurements of pore size were conducted on randomly selected pores and imaged at different magnifications. Hydrogels without PA presented irregularly arranged pores, with smaller ones at the edge and larger ones at the center (Figure 6(a1,a2)). The pore size ranged from about 30 to 100 μm.

Hydrogels with lower amounts of F-127 and PA (Figure 6(b1,b2,c1,c2)) present disorganized pores of varying sizes, showing thick boundaries between them (Figure 6(b1,b2)) or a rough surface with fewer pores than a, b, and d (Figure 6(c1,c2)). Hydrogels with higher amounts of F-127 and PA present a highly porous and inter-connected hydrogel (Figure 6(d1–d4)). The incorporation of PA thus led to a more densely interconnected polymer network between F-127 and G, yielding hydrogels with increased and uniform pore formation compared to other samples.

### 2.4. Thermal Analysis

**Figure 7:**
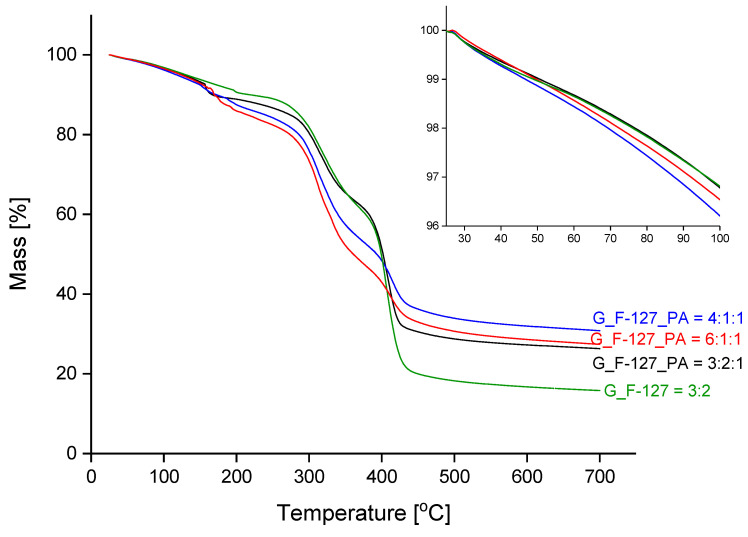
Dynamic TG curves of the prepared samples under an inert atmosphere. Inset: magnification of the temperature ranges from 25 °C to 100 °C.

**Figure 8:**
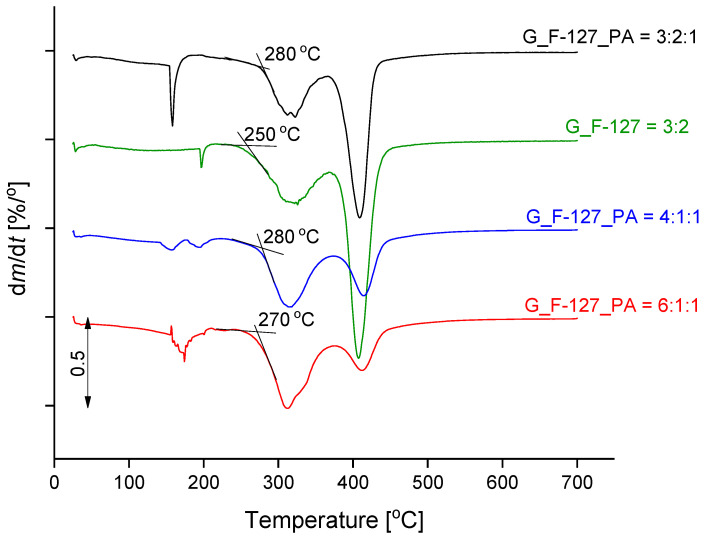
DTG curves of the investigated xerogel samples.

The course of the thermal decomposition of the prepared xerogel samples with different formulations is shown in Figure 7, and the corresponding derivative curves (DTG) are presented in Figure 8. The inset graph in Figure 7 shows an enlargement of the temperature range between 25 °C and 100 °C; the mass loss within this range is summarized in Table 2.

During the first step of the mass loss curve, from room temperature to about 250 °C, a dehydration process occurs. The abrupt mass loss from 150–200 °C is attributed to the evolution of water molecules retained in the closed pores. In contrast, freeze-dried samples do not show this behaviour, as the water was removed from the pores in the final preparation step (see Supplementary Figures S1 and S2). The mass loss up to 100 °C was similar for all four samples and is around 3.5%. The value most likely depends on the relative humidity of the environment. For the lyophilized samples, the mass loss in this range is highest for the sample with uniform pore distribution, G_F-127_PA = 3:2:1 (7.0%; Supplementary Table S1). The thermal decomposition of the organic substance takes place at temperatures above 250 °C in two successive steps that overlap. The DTG curves (Figure 8) show that the addition of phytic acid increases the thermal stability; the sample G_F-127 = 3:2 begins to decompose at 250 °C, while the other samples with added PA are 20 to 30 degrees higher. The addition of cross-linkers reduces the rate of thermal decomposition in the last step, from about 370 °C, and increases the mass of the solid residue at the final temperature. One of the reasons for the latter finding is that the PA is added in the form of sodium salt. The results for the corresponding lyophilized samples show the same behavior (Supplementary Figures S1 and S2).

**Figure 9:**
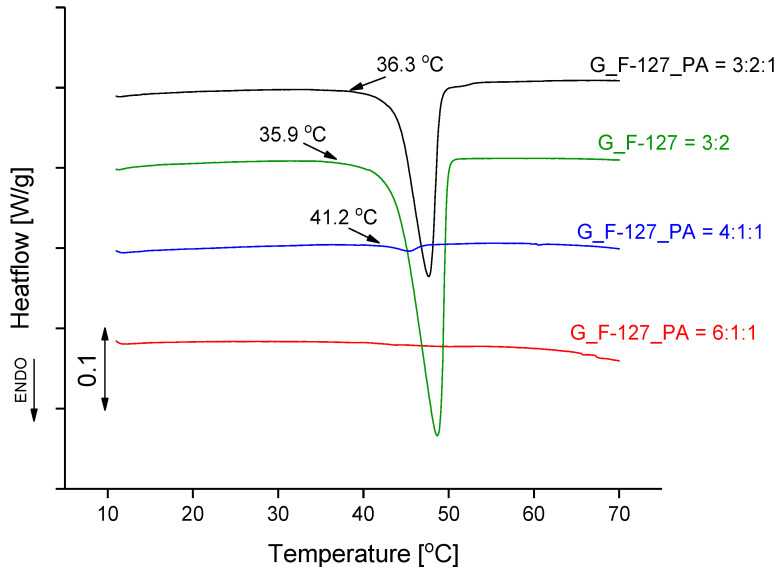
DSC curves of the investigated xerogel samples.

DSC curves (Figure 9) in the range from 10 °C to 70 °C show that the melting temperature of sample G_F-127_PA = 3:2:1 is very similar to the melting temperature of sample G_F-127 = 3:2, while the enthalpy of the process is nearly halved. The other two samples show no significant melting effect (Table 3). Although the initial temperatures of the melting endotherm are above human body temperature, the deviation from the baseline starts at a temperature of less than 37 °C in both cases. The exposure of hydrogel to this temperature results in melting. For the lyophilized samples, the endothermic melting peaks are shifted to higher temperatures of 5 to 7 degrees (Supplementary Figure S3 and Table S2); the melting enthalpies are also different but show the same trend. 

**Figure 10:**
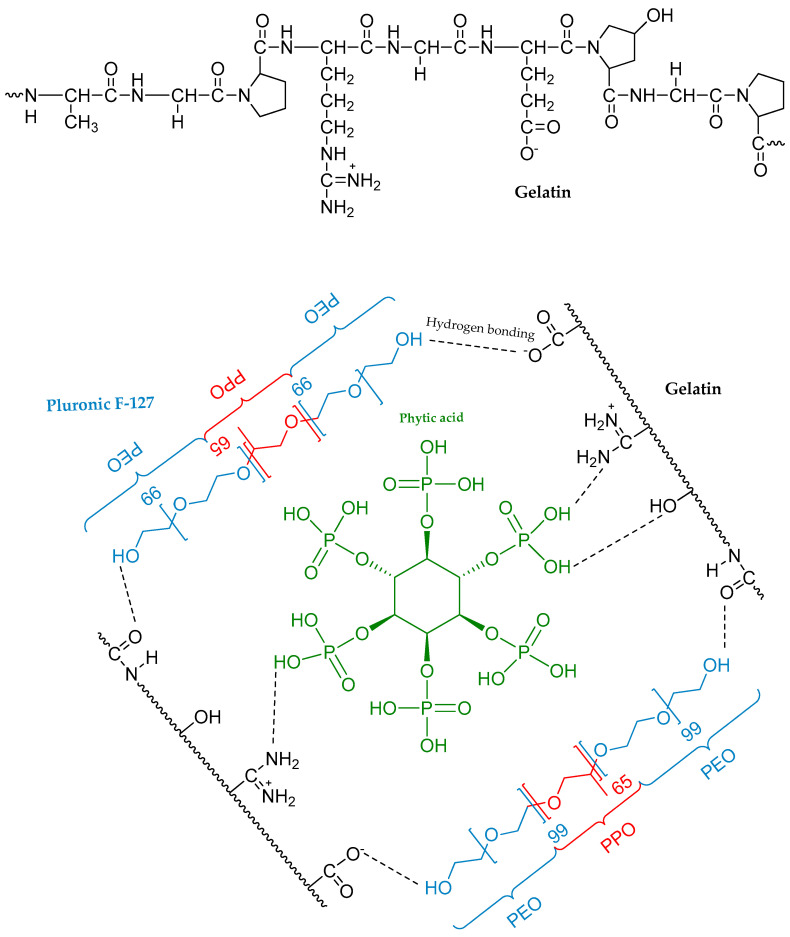
Schematic illustration of the partial process involved in hydrogel formation based on gelatine, phytic acid, and Pluronic F127, consisting of poly(ethylene oxide)-b-poly(propylene oxide)-b-poly(ethylene oxide) (PEO-PPO-PEO).

**Figure 11:**
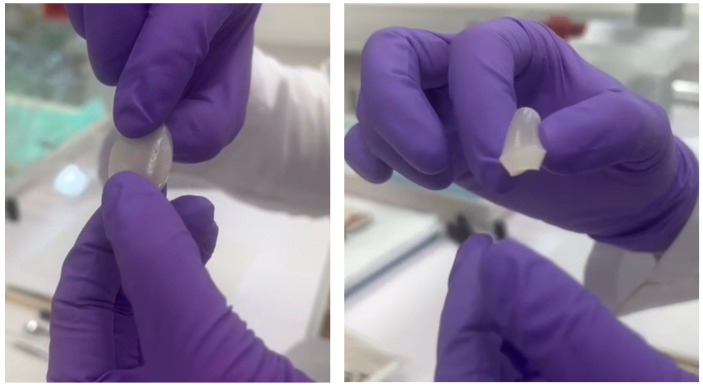
Elasticity assessment of hydrogel (G_F-127_PA = 3:2:1).

The inclusion of PA in the mixture facilitated the development of hydrogels stabilized through intermolecular bonds between the functional groups of all compounds [24], schematically presented in Figure 10. Pluronic F-127 was incorporated into gelatin, in which the chains contain numerous functional groups capable of forming hydrogen bonds. Consequently, the gelatin chains can be readily cross-linked in solution using phytic acid, which is rich in phosphate groups. Remarkably, the increasing amounts of F-127 and PA lead to intensified hydrogen bonding and other electrostatic interactions between the components, influencing the stability of the hydrogel network (Figure 11). Overall, the incorporation of phytic acid enhances the cross-linking efficiency of the hydrogel network, offering promising avenues for the development of advanced biomaterials with enhanced performance and functionality.

The Video S1 demonstration of the elasticity assessment of hydrogel is provided in the Supporting Materials.

## 3. Conclusions

In summary, this study introduced the synthesis of advanced hydrogels by physical cross-linking of gelatin, Pluronic F-127, and phytic acid. The properties of hydrogels depend on the ratio between these three components. As the content of F-127/PA increases, the obtained hydrogel demonstrates enhanced properties. Furthermore, the study highlights the importance of optimizing the composition of hydrogels to achieve desired properties, emphasizing the role of Pluronic F-127 and phytic acid in enhancing stability. The most stable hydrogel was achieved by incorporating a higher amount of F-127 and PA (G_F-127_PA = 3:2:1). These findings were also supported by thermal analysis, which indicated higher stability for the hydrogel (G_F-127_PA = 3:2:1) when compared to other hydrogel samples. The intermolecular bonding between the three components was observed in the FTIR spectra through the shifts in absorption bands specific to the hydroxyl groups. All hydrogels demonstrated favorable swelling properties. The hydrogel (G_F-127_PA = 3:2:1), containing higher amounts of both phytic acid and Pluronic F-127, showed reduced swelling in acidic conditions (587%) and increased swelling in PBS (pH 7.4) at 1544%. Overall, the hydrogel with a higher amount of F-127/PA was more stable and achieved a higher degree of swelling. The results were also supported by TD/DSC analysis. SEM microscopy evidenced a highly porous network with fine and interconnected pores, especially in the case of sample G_F-127_PA = 3:2:1. This not only indicates their ability to absorb a significant amount of water but also highlights their potential as versatile and biocompatible materials with promising properties for advanced applications in biomedicine and beyond.

Future work will involve the application of these innovative hydrogels for wound healing purposes.

## 4. Materials and Methods

### 4.1. Chemicals and Materials

Gelatin from bovine skin, phytic acid sodium salt hydrate, and Pluronic^®^ F-127 were purchased from commercial sources Sigma-Aldrich (Sigma Aldrich, St. Louis, MO, USA; and Sigma-Aldrich Chemie GmbH, Steinheim, Germany, Merck, Darmstadt, Germany) without additional purification processes. The heating of mixtures was performed in a Mettler-Toledo Easymax 102 Advanced Synthesis Workstation using 25 mL closed reactor tubes.

### 4.2. Sample Preparation

Various ratios of gelatin, Pluronic F-127, and phytic acid were examined to determine the optimal conditions for hydrogel formation, as detailed in Table 1. A mixture of gelatin, F-127, and phytic acid was placed into 25 mL reactor tubes, followed by the addition of MilliQ water. The vial was tightly sealed, heated to 65 °C, and continuously stirred (400 rpm) for one hour to achieve a uniform solution. The resulting mixture was transferred to a microplate, and the hydrogels were allowed to form at room temperature. The prepared hydrogels were further lyophilized or dried under room conditions (xerogels) for characterization.

### 4.3. Characterization

#### 4.3.1. FTIR Spectroscopy

The Fourier-transform infrared (FTIR) spectra of the initial reagents phytic acid, gelatin, and copolymer Pluronic F-127 (serving as a reference), along with the optical xerogel, were recorded utilizing a PerkinElmer Spectrum 100 instrument (Waltham, MA, USA) equipped with universal attenuated total reflection (UATR) sampling accessory. Spectra were obtained in the 4000 to 600 cm^−1^ range, employing a resolution of 4 cm^−1^ and an average of four scans.

#### 4.3.2. Swelling Experiments

The air-dried hydrogels underwent a swelling capacity assessment. This involved immersing the hydrogels in various media, including phosphate buffer (Na_2_HPO_4_, KH_2_PO_4_) at pH 6.0 and 8.0, phosphate-buffered saline solution (0.1 M PBS, pH = 7.4), and SM buffer. The samples were weighed periodically over 74 h. Following this procedure, the swollen hydrogels were extracted, excess liquid on the surface was removed using filter paper, and the weight was measured. The swelling degree (Sw) of the sample was then determined using the following calculation:

Sw = (W_t_ − W_0_)/W_0_ × 100 (1)

where W_0_ and W_t_ are the weights of the dry and wet samples, respectively.

#### 4.3.3. Scanning Electron Microscopy (SEM) Investigation

SEM examinations were performed on lyophilized hydrogels obtained under consistent conditions with respect to freezing time, method, vacuum pressure, temperature, and other relevant parameters, to elucidate potential morphological distinctions. The lyophilized hydrogel was cut with a sharp knife into a square just a few millimeters in size. The samples were sputter-coated with a carbon layer, using a BAL-TEC SCD 005 sputter coater. The coating surface appearance along the cross-section was evaluated using field emission scanning electron microscopy (FEI Helios NanoLab 600 Dual-beam, Hillsboro, OR, USA). Imaging was performed by SEM using secondary electron imaging (SEI) mode at 5 kV.

#### 4.3.4. Thermal Analysis

TG measurements were performed on the Mettler Toledo TGA/DSC1 instrument (Columbus, OH, USA) in a temperature range from 25 to 700 °C. The heating rate was 10 K/min. During the measurement, the furnace was purged with argon with a flow rate of 100 mL/min; 150 µL platinum crucibles were used, and the initial masses of the samples were between 6 and 10 mg. Lyophilized samples in the form of thick films were cut to a suitable dimension using scissors. In all measurements, the blank curve was subtracted.

For studying the melting properties of the prepared samples, DSC measurements were carried out separately on a Mettler Toledo DSC1 instrument. Samples were carefully weighed on external Mettler Toledo MX5 balance in 40 μL pans and covered with lids. The temperature range was from 10 °C to 70 °C, and the heating rate was 2 K/min. Empty Al pan served as a reference. The furnace was purged with argon with a flow rate of 100 mL/min. Masses of samples used were similar to TGA measurements, around 5 mg. The DSC analyzer was calibrated with MilliQ water and high-purity indium from Mettler Toledo.

## Figures and Tables

**Table 1:** The weights of compounds used to prepare hydrogels and the sample codes. Reaction condition: T = 65 °C; t = 1 h.

Entry	Gelatin (g)	F-127 (g)	Phytic Acid (g)	Sample Code
1	0.2	0.05	0.05	G_F-127_PA = 4:1:1
2	0.3	0.05	0.05	G_F-127_PA = 6:1:1
**3**	**0.3**	**0.2**	**0.1**	**G_F-127_PA = 3:2:1**
4	0.3	0.2	/	G_F-127 = 3:2

**Table 2:** Comparison of the mass loss of hydrogels from room temperature to 100 °C.

Δ*m* (25–100 °C)/%			
G_F-127_PA = 4:1:1	G_F-127_PA = 6:1:1	G_F-127_PA = 3:2:1	G_F-127 = 3:2
3.79	3.46	3.22	3.19

**Table 3:** Melting temperatures and the corresponding enthalpies of the xerogel samples.

Sample	Onset Melting Temperature/°C	Δ*H*/J g^−1^
G_F-127_PA = 4:1:1	42.9	0.60
G_F-127_PA = 6:1:1	/	/
G_F-127_PA = 3:2:1	43.9	23.9
G_F-127 = 3:2	43.8	40.0

## Data Availability

All data and materials are available on request from the corresponding author. The data are not publicly available due to ongoing research using part of the data.

## Supplementary Materials

The following supporting information can be downloaded at: https://www.mdpi.com/article/10.3390/gels10050294/s1, Figure S1. Dynamic TG curves of the lyophilized samples. Inset: the enlargement of the temperature range from 25 °C to 100 °C. Table S1. Comparison of the mass loss of lyophilized hydrogels from room temperature to 100 °C. Figure S2. DTG curves of the lyophilized samples. Figure S3. DSC curves of the lyophilized samples. Table S2. Melting temperatures and the corresponding enthalpies of the lyophilized samples. Video S1, Elasticity assessment of hydrogel (G_F-127_PA = 3:2:1).
