# Supplementary material for: Development and Characterization of Gelatin-Based Hydrogels Containing Triblock Copolymer and Phytic Acid
Source: Gels. 2024 Apr 25;10(5):294. doi: 10.3390/gels10050294 (PMC11121302; doi:10.3390/gels10050294)
Supplement: Supplementary file 1 [file gels-10-00294-s001.zip › gels-2983019-Supplementary.pdf]

Supplementary Materials

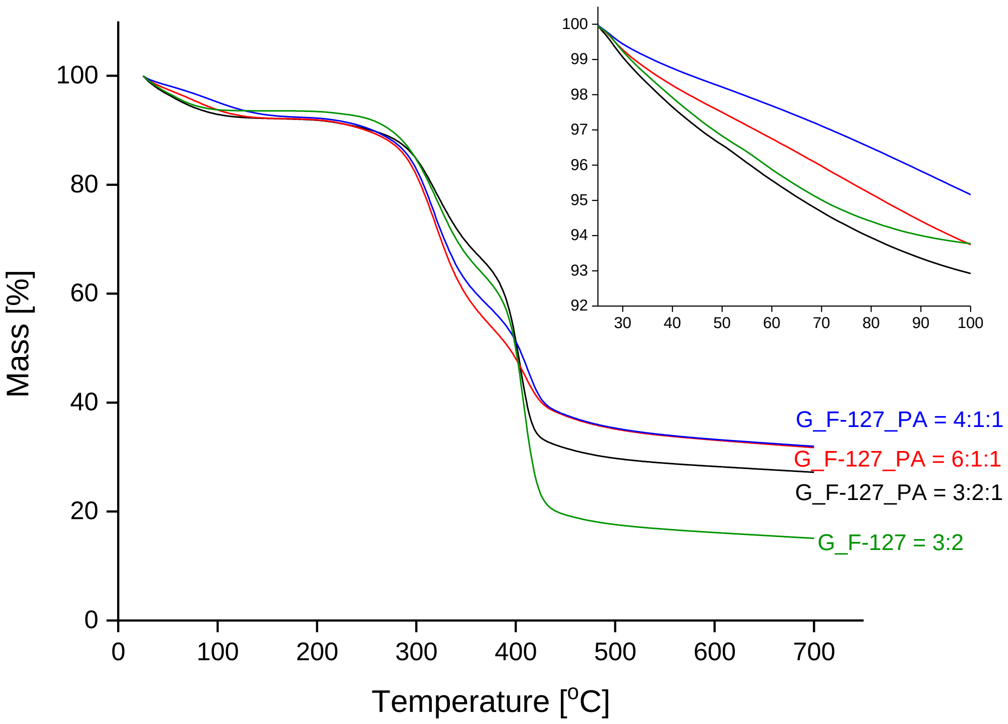

Figure S1. Dynamic TG curves of the lyophilized samples. Inset: the enlargement of the temperature range from 25 °C to 100 °C.

Table S1. Comparison of the mass loss of lyophilized hydrogels from room temperature to 100 °C.

Δ*m* (25–100 °C) / %			
**G_F-127_PA = 4:1:1**	**G_F-127_PA = 6:1:1**	**G_F-127_PA = 3:2:1**	**G_F-127 = 3:2**
4.83	6.25	7.07	6.22

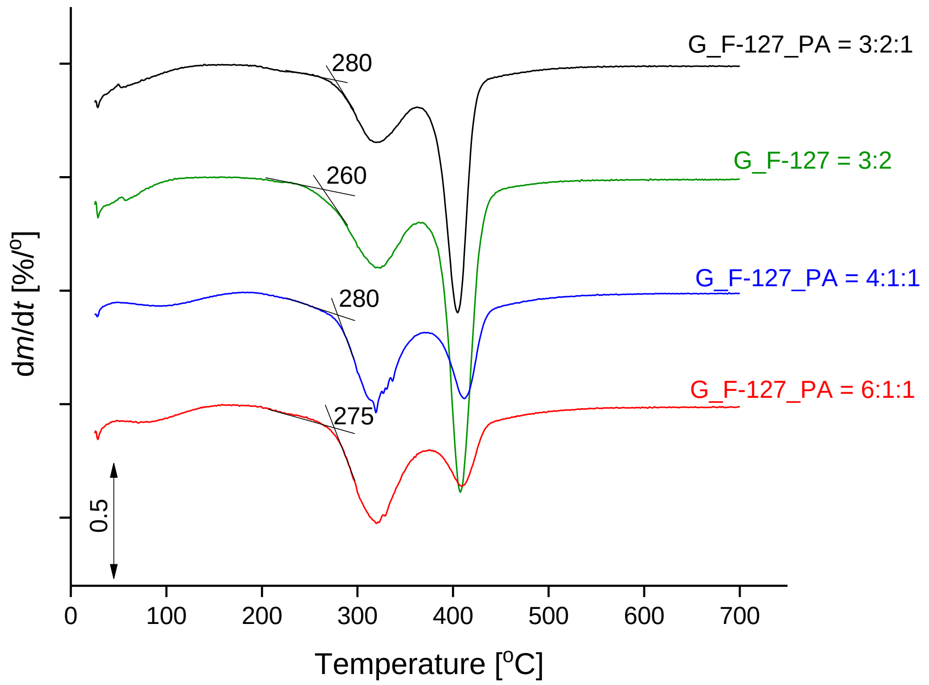

Figure S2. DTG curves of the lyophilized samples.

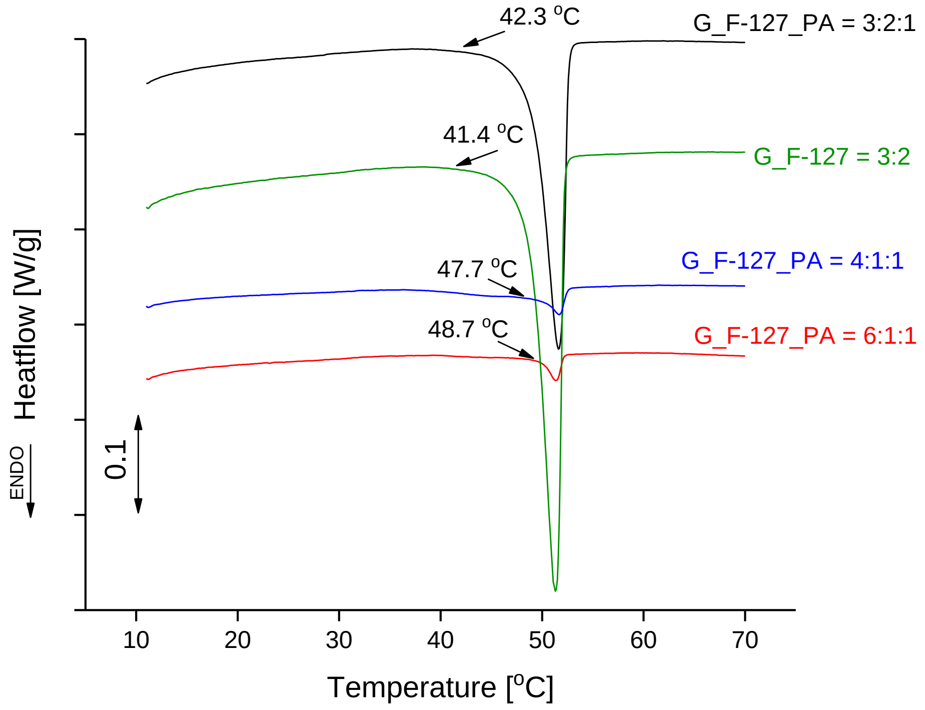

Figure S3. DSC curves of the lyophilized samples.

Table S2. Melting temperatures and the corresponding enthalpies of the lyophilized samples.

sample	Onset melting temperature / °C	ΔH / J g^{-1}
G_F-127_PA = 4:1:1	49.6	2.77
G_F-127_PA = 6:1:1	49.9	1.28
G_F-127_PA = 3:2:1	48.9	26.7
G_F-127 = 3:2	48.8	34.2
