# Supplementary figures and images for: Development and Characterization of Gelatin-Based Hydrogels Containing Triblock Copolymer and Phytic Acid
Source: Gels. 2024 Apr 25;10(5):294. doi: 10.3390/gels10050294 (PMC11121302; doi:10.3390/gels10050294)

## Slide 1
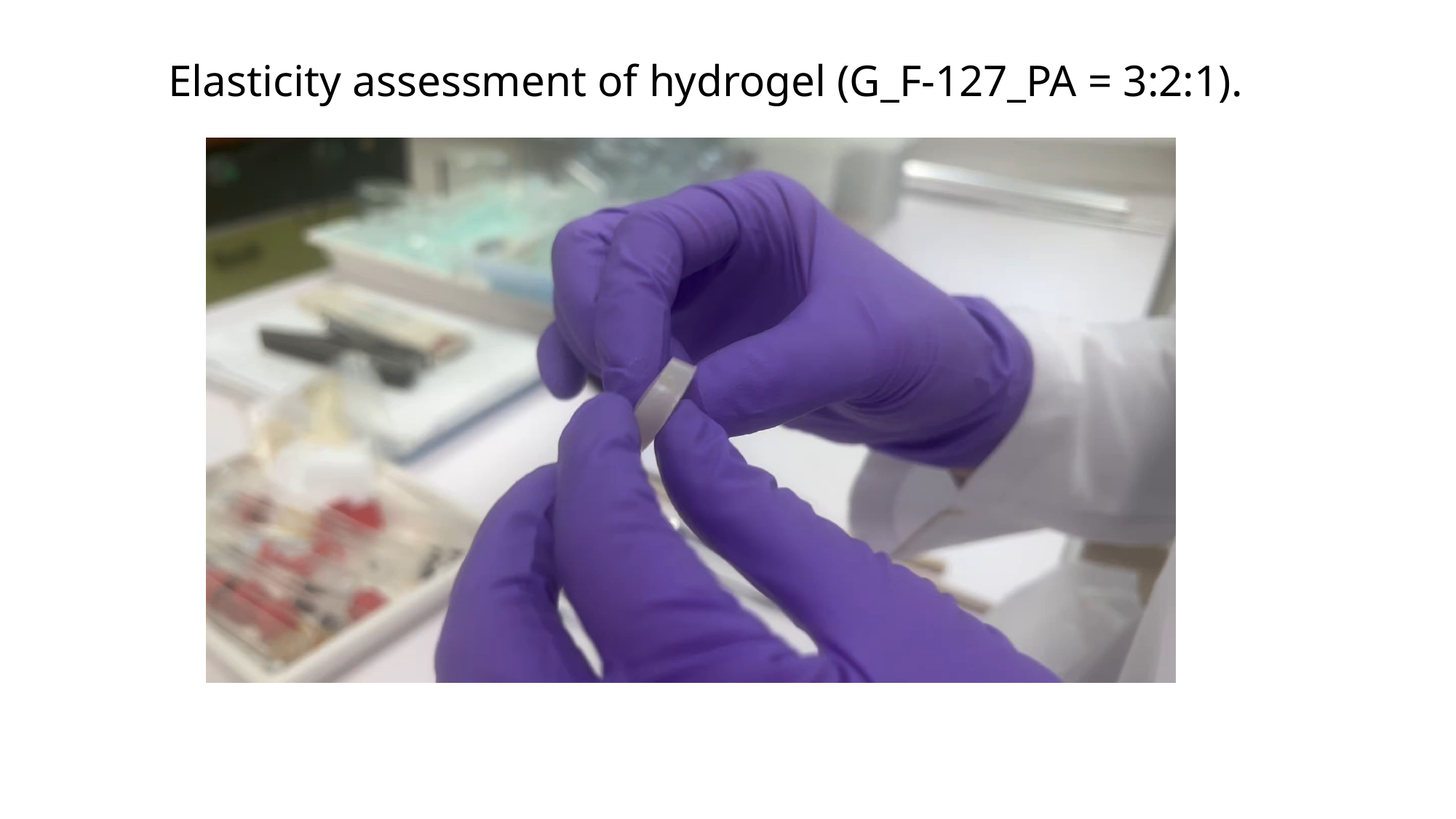

Elasticity assessment of hydrogel (G_F-127_PA = 3:2:1).

Supplement: Supplementary file 1 [file gels-10-00294-s001.zip › gels-2983019-Video S1.pptx]
